# Developmental cascades from early childhood attachment security to adolescent level of personality functioning among high-risk youth

**DOI:** 10.1017/S0954579424001044

**Published:** 2024-06-27

**Authors:** Emily T. O’Gorman, Gregory J. Meyer

**Affiliations:** 1 Department of Psychiatry and Behavioral Sciences, Johns Hopkins School of Medicine, Baltimore, MD, USA; 2 Department of Psychology, University of Toledo, Toledo, OH, USA

**Keywords:** attachment, adverse childhood experiences, developmental cascades, genotype, level of personality functioning

## Abstract

This study examines associations between early childhood attachment security and adolescent personality functioning in a high-risk sample within a developmental psychopathology framework. Data from 2,268 children (1165 male; 1103 female) and caregivers participating in Future of Families and Child Well-Being Study (FFCWS) were used to examine (1) effects of genetic polymorphisms of the serotonin transporter (5-HTTLPR) and dopamine D4 receptor (DRD4) genes and adverse childhood experiences (ACEs) on attachment security and emotional and behavioral dysregulation in early childhood and (2) longitudinal associations and transactional relationships among attachment security, dysregulation, negative parenting attitudes and behaviors, social competence, and adolescent personality functioning. Results revealed that ACEs predicted attachment security over and above sex or the genetic risk, and gene × environment interactions did not increment prediction. Results of cascade models showed that greater early childhood attachment security predicted higher adolescent level of personality functioning via pathways through intermediary variables. Limitations and future research directions are discussed.

A lifespan developmental approach to personality and psychopathology recognizes the important roles of childhood antecedents and learning history. There is widespread recognition that the quality of relationships with the self and others impacts development toward positive adaptation or pathology (e.g., Sharp & Wall, 2018). The importance of this construct is evident in its inclusion in the Alternative Model for Personality Disorders (AMPD; American Psychiatric Association, 2013) as Criterion A, or Level of Personality Functioning (LPF). LPF reflects severity of personality pathology and assesses four separate but interrelated constructs in the domains of self and interpersonal functioning: identity and self-direction in the former, and empathy and intimacy in the latter. LPF domains span both normal and pathological personality functioning and are relevant to all forms of psychopathology to some degree (Bender, 2019).

While not explicitly designated a developmental continuum, the operationalization of LPF was influenced by developmental theories. The result is a continuum of severity of impairment in personality functioning that parallels the normative developmental task of identity formation (Sharp & Wall, 2018). Developmental psychopathology and, more specifically, developmental cascades models offer an ideal framework within which to examine this process. Developmental cascades refer to the cumulative consequences of these dynamic transactions among developing systems over time (Masten & Cicchetti, 2010). Cascade effects have been used to illustrate probabilistic links between multiple antecedent factors and specified outcomes as well as a fanning out effect, whereby functioning in one domain affects functioning in other domains over the course of development. Cascade models of development enable researchers to examine separate but interrelated causal processes in order to identify factors that promote or undercut adaptive or pathological functioning (Masten & Cicchetti, 2010). The present research utilized this framework to examine personality developmental outcomes as the result of dynamic interplay between neurobiological factors, social and environmental feedback, and internal psychological experiences (Crowell et al., 2009). More specifically, this project examined developmental pathways to LPF, incorporating multiple levels of analysis – cellular, individual, and environmental – and assessing separate but interrelated constructs – self-regulation, caregiver-child interactions, and social competency – at multiple time points between infancy and adolescence.

## Attachment organization

Attachment theory (Bowlby, 1982) is concerned with the formation and course of biologically based patterns of behavior within significant relationships (e.g., child-caregiver). While early experience and developmental context play causal roles in the emergence and course of attachment organization, context also evolves through ongoing transactions between the child and their environment. Thus, attachment patterns start forming in infancy and are shaped by temperament, self-regulation, and other internal psychological characteristics (Sroufe et al., 1999) as well as by interactions with caregivers, peers, and romantic partners throughout the lifespan (Ainsworth, 1989). Importantly, research indicates that attachment patterns are predictive of positive adaptation or pathology in various domains, with insecure attachment in early childhood and throughout development is linked to impaired emotional regulation and social competence and is probabilistically associated with later development of psychopathology (Sroufe, 2005).

## Emotional and behavioral regulation

Personality maturation involves increasing organization of self-regulatory processes, including modulation of emotional reactivity and control of behavioral responses (Westen, 1994). Emotional and behavioral dysregulation have been shown to be transdiagnostic factors associated with development of general psychopathology and personality pathology (Aldao et al., 2016; Stepp et al., 2016) and to be predictive of social and psychological maladjustment across the lifespan (Cain et al., 2019; Calkins & Fox, 2002). Evidence suggests the capacity for self-regulation of emotions and behaviors is shaped by the early caregiving environment and develops concurrently with self and interpersonal functioning (Ainsworth, 1989; Kaufman & Crowell, 2018). Within the context of dyadic attachment relationships, caregivers’ responsivity to children’s needs scaffolds development of regulatory strategies (Sroufe, 2005). Over time, the consistency with which a caregiver accurately appraises and appropriately responds to a child’s needs informs the child’s confidence in their capacity to self-regulate. Conversely, inconsistency – either in a caregiver’s responsivity or one’s own responses to contextual demands – is linked to disturbances in self and interpersonal functioning (Ainsworth, 1989) and can be viewed as a marker of early pathological processes that are probabilistically associated with later pathology (Sroufe, 2005). Importantly, longitudinal research has shown that self-regulation interacts with both caregiver and peer relationships across childhood, predicting higher levels of psychopathology (Kim & Cicchetti, 2010) and personality pathology (Hallquist et al., 2015) in adolescence.

## Molecular genetics and neurobiology

A primary aim of research on molecular genetics has been identifying genetic variants that confer vulnerability to pathological personality traits. The serotonergic and dopaminergic systems impact cognitive and affective processing and have been implicated in the development of personality pathology; as such, researchers have been particularly interested in genetic variants of the serotonin transporter (*5-HTT*) and dopamine D4 receptor (*DRD4*) genes (Beauchaine et al., 2009).

### Serotonin transporter (5-HTT)

Serotonin plays important roles as a trophic factor in neural development and as a neurotransmitter regulating cognitive processes (e.g., attention), emotion and behavior, and social cognition (Brummelte et al., 2017; Hariri & Holmes, 2006). Serotonergic activity has also been implicated in expression of normal and pathological personality traits (Skodol et al., 2002; Takano et al., 2007) and interpersonal style (Tse & Bond, 2002). Research has indicated that individual differences in serotonergic activity are dictated in part by genetic variation in the gene that codes for the serotonin transporter (*SLC6A4*; Brummelte et al., 2017). The linked polymorphic region of the serotonin transporter gene (*5-HTTLPR*) is tri-allelic. The short allele (S) and functionally equivalent long G-allele (*L*_G_) have lower transcriptional efficacy and have been associated with higher levels of neuroticism, increased emotional reactivity, and increased risk of psychopathology compared with the long A-allele (*L*_A_) (Auerbach et al., 2001; Homberg & Lesch, 2011).

Meta-analytic findings indicate the direct effect of *5-HTTLPR* on personality and psychopathology is small at best (Minelli et al., 2011). Rather, the relationship between *5-HTTLPR* and manifest psychopathology may be moderated by multiple factors, including sex (Gressier et al., 2016), race (Gelernter et al., 1999), life stress (Gressier et al., 2016), and caregiver responsiveness (Barry et al., 2008; Spangler et al., 2009), among other factors. Cicchetti et al. (2011) examined the impact of genotype on attachment style in children of low-income families exposed versus not exposed to child maltreatment. Findings revealed that the *5-HTTLPR* S allele (S/S or S/L) conferred increased risk of attachment insecurity and disorganization at age two, but only for children who had not experienced maltreatment (Φ = .38). Among maltreated children, risk of attachment disorganization was high regardless of genotype (Cicchetti et al., 2011).

### Dopamine D4 receptor (DRD4)

The dopaminergic system has also received attention for its role as a moderator of several psychological processes, including motivation, attention, and reward processing (Girault & Greengard, 2004). In particular, evidence suggests the dopamine D4 receptor (*DRD4*), which is localized primarily in the prefrontal cortex and amygdala, is associated with emotional processing and encoding of emotionally salient associative memories (Lauzon & Laviolette, 2010). Variation across individuals in *DRD4* function has been linked to a polymorphism of the gene coding for *DRD4* involving a 48 base pair variable number of tandem repeats (*VNTR*) in the exon 3 region (Wang et al., 2004). Research indicates the seven-repeat (7R) allele, relative to the four-repeat (4R) allele, results in reduced *DRD4* efficiency and is associated with increased impulsivity and novelty-seeking, higher levels of negative affect, and socioemotional dysfunction (Holmboe et al., 2011; Pappa et al., 2015).

Research indicates *DRD4 VNTR* expression varies based on sex (Dmitrieva et al., 2011; He et al., 2018). Meta-analytic findings suggests that the presence of at least one *DRD4* 7R allele is associated with differential susceptibility to adverse and supportive rearing environments (*r* = .37; Bakermans-Kranenburg & Van Ijzendoorn, 2011). For example, research indicates the impact of caregiving quality on children’s self-regulatory capacity is greater for children with the 7R allele (Sheese et al., 2007; Windhorst et al., 2015). Moreover, in a randomized controlled trial of an intervention program for promoting positive parenting, children with the 7R allele benefited more from intervention-related changes in parenting behavior (Bakermans-Kranenburg et al., 2008).

Several studies have found that *DRD4* allelic variation is significantly associated with attachment; however, directions of the effects are inconsistent, suggesting environmental factors moderate the impact of *DRD4 VNTR* variation on attachment (Golds et al., 2020). The findings of Cicchetti et al. (2011) supported the importance of attending to environmental risk factors. Specifically, findings indicated that the presence of at least one *DRD4* 7R allele was associated with increased risk of attachment insecurity and disorganization at age two among children who had not experienced maltreatment (Φ = .38) but not among children who had experienced maltreatment (Cicchetti et al., 2011).

#### HTT and DRD4

Gene expression is moderated not only by exogenous environmental factors but also by endogenous environmental factors, including physiological responses, neural activity, and expression of other genes (Gottlieb, 2007). Studies examining epistasis between *5-HTTLPR* and *DRD4 VNTR* polymorphisms in predicting attachment have had mixed results. Lakatos et al. (2003) found that infants from a low-risk population with at least one copy of both the *5-HTTLPR* L and *DRD4* 7R alleles exhibited lower negative emotionality when approached by a stranger in the Strange Situation procedure; however, the interaction between the two alleles for predicting attachment was not significant. Consistent with this, the three other studies (Leerkes et al., 2017; Luijk et al., 2011; Spangler et al., 2009) found non-significant interactions between *5-HTTLPR* and *DRD4 VNTR* polymorphisms for predicting attachment classification. Cicchetti et al. (2011) did not examine interactions between candidate genes directly. However, a composite risk variable defined by the presence of one or both risk genotypes (*5-HTTLPR* S/S or S/L and/or *DRD4* 7R) significantly predicted attachment classification among non-maltreated children at age two (Φ = .50). Moreover, the frequencies of *5-HTTLPR* and *DRD4* polymorphisms among non-maltreated children with secure versus insecure attachment classifications appear to be consistent with the findings of Lakatos et al. (2003). Among non-maltreated children, the majority (75%) of those with secure attachment were homozygous for the long *5-HTTLPR* allele (L/L), suggesting that this may protect against development of insecure or disorganized attachment.

## Present study

Research conducted within a developmental cascades framework presents methodological challenges, most notably due to the need for longitudinal data and for data collected across multiple levels of analysis (Masten & Cicchetti, 2010). As a result, few studies have employed this methodology. The current project addresses this gap in the literature by incorporating multiple levels of analysis – genetic, psychological, and interpersonal – and assessing separate but interrelated constructs – regulation, parenting, and social competence – across multiple time points using data from The Future of Families and Child Wellbeing Study (FFCWS; Reichman et al., 2001). The use of the FFCWS sample, which is comprised of low-income urban families, is significant, because it allows for examination of the impact of proximal processes on individual differences in conditions of greater instability.

Three primary hypotheses were advanced. First, it was expected that genetic and environmental risk would interact to predict attachment security and emotional and behavioral dysregulation in early childhood. Second, it was expected that dysregulation, negative parenting, and social competency would be mutually reinforcing, such that dysregulation and negative parenting would increase, and social competence would decrease, or vice versa, over time. Finally, it was expected that attachment security in early childhood would initiate a developmental cascade, influencing personality functioning in adolescence via regulation, parenting attitudes and behaviors, and social competence across early and middle childhood.

## Method

### Participants

Participants included children and primary caregivers from the FFCWS (Reichman et al., 2001). Of the total 4,898 children in the FFCWS sample, 2,268 children (1165 male; 1103 female) and caregivers were included in the present sample. The majority (98.8%) of primary caregivers were children’s biological mothers, followed by biological fathers, maternal or paternal grandmothers, other relatives, or non-relatives (e.g., foster parents). Cases were included if they had data available for attachment style assessed during the in-home assessment at age three. The sample was predominantly Black (55.5%), with 27.4% identifying as White and 17.1% identifying as another race. Socioeconomic characteristics of the present sample were comparable to that of the full FFCWS sample; 77.7% of children had single mothers, 34.7% of mothers had less than high school education or equivalent, and 34.3% of families had household incomes below the poverty line. Demographic characteristics of the present sample, full FFCWS sample, and U.S. population at baseline are contained in the Appendix.

### Procedures

Data were collected at birth and ages one, three, five, nine and 15 (Y0, 1, 3, 5, 9, and 15, respectively). Each wave consisted of interviews with children’s biological parents and/or primary caregivers. Data collection at Y1 and later included in-home assessments of a subsample. Data collection at Y9 and Y15 included child interviews. Table 1 contains measures used in the present study and timing of their administration.

### Methods and measures

#### DNA collection and extraction

DNA was collected through saliva samples at Y9 using Oragene^®^ DNA Self-Collection Kits. DNA was purified and extracted using a standardized centrifugation procedure. Genotyping was conducted following established protocols (Bendheim-Thoman Center for Research on Child Wellbeing, 2018).

#### Adverse childhood experiences (ACEs)

Exposure to ACEs for the first three years of life was quantified using parent-report at ages 1 and 3. Nine of the ten ACEs included in the Center for Disease Control and Prevention Kaiser ACE study (Felitti et al., 1998) were assessed: four categories of child maltreatment (i.e., physical abuse, psychological abuse, emotional neglect, physical neglect) and five categories of household dysfunction (i.e., substance use, mental illness, domestic violence, incarceration, and parental separation or divorce). Information on sexual abuse was not available. Prior research (e.g., Felitti et al., 1998) has typically treated ACEs categories as dichotomous variables (i.e., exposure vs. no exposure). However, to avoid loss in effect size, power, and measurement reliability (MacCallum et al., 2002), dimensional data were retained in the present study. This was accomplished by computing between-subjects *z*-scores for each ACE category at Y1 and Y3, averaging *z*-scores across categories within years for each participant, and then taking the average of the *z*-scores for ages 1 and 3. Internal consistency across ACE categories was acceptable (α_Y1_ = .63; α_Y3_ = .71).

#### Attachment

The Toddler Attachment Q-Sort (TAS-39; Andreassen & Fletcher, 2007) was completed during in-home interviews with mothers and their children at age 3. Interviewers directed mothers to sort 39 cards containing child characteristics or behaviors relevant to attachment based on their relative frequencies. TAS-39 data were analyzed as described in Bimler and Kirkland (2002) to identify attachment classifications, resulting in 75.8% of the sample classified as securely attached, 22.1% as insecure-resistant, and 2.1% as insecure-avoidant. A continuous variable reflecting the distance between each child’s specific profile and the secure attachment classification was then derived. This variable was then multiplied by −1 to reverse the scale, thus quantifying each child’s degree of attachment security (i.e., higher values indicate greater security); the attachment security variable was used in the primary analyses in the present study.

#### Dysregulation

Modified versions of the Child Behavior Checklist (CBCL; Achenbach & Rescorla, 2001) were administered in the FFCWS to mothers when children were 3, 5, 9, and 15. In the present study, emotional and behavioral dysregulation was assessed using the CBCL Dysregulation Profile (CBCL-DP; Ayer et al., 2009), which is defined by elevations on the Aggressive Behavior, Anxious/Depressed, and Attention Problems scales. Because the number of items administered varied across-time points, standardized scores (*M* = 0, *SD* = 1) were used, with higher scores indicating higher levels of behavior problems.

#### Negative parenting attitudes and behaviors

A composite measure of negative parenting attitudes and behaviors was derived from 15 CTSPC (Straus et al., 1998; Straus, 2004) items and four Aggravation in Parenting (Hofferth et al., 1997) items, which mothers completed at Y3, Y5, Y9, and Y15. To measure chronicity of negative parenting behaviors, CTSPC items were recoded by assigning weights to values in accordance with the frequencies indicated by the response categories (0 = never; 1 = yes, but not in the past year; 2 = once; 3 = twice; 4 = 3–5 times; 8 = 6–10 times; 15 = 11–20 times; 25 = more than 20 times). Internal consistency for Negative Parenting was acceptable (αs = .70, .69, .75, and .64 for Y3, 4, 5, and 6, respectively).

#### Social competence

Social competence was assessed with 10 items from the Express subscale of the Adaptive Social Behavior Inventory (ASBI; Hogan et al., 1992), which mothers completed at Y3, Y5, and Y9. Three items were omitted, because they were included in a parallel self-report form that was completed by focal children at age 15, which contributed to the LPF outcome variable.

#### Level of personality functioning (LPF)

LPF was derived from six self-report and six parent-report indicators completed at Y15. A self-report composite was formed using five subscales of the EPOCH Measure of Adolescent Wellbeing (Kern et al., 2016) and a self-report adaptation of the ASBI (α = .84). The EPOCH includes five dimensions: Engagement, Perseverance, Optimism, Connectedness, and Happiness. The parent-report composite included six CBCL items with content reflecting aspects of LPF as they are delineated in the DSM-5 AMPD. These items were not included in the CBCL-DP to avoid overlap between the predictor and criterion variables. *Z*-transformations were applied to the two composites, and LPF was computed as the average of the two *z-*scores.

### Primary data analyses

Hierarchical linear regression analyses were used to determine whether genotype and environmental risk (i.e., ACEs) interact to predict attachment security and dysregulation. Six models were constructed to test all possible combinations of the three risk genotypes (i.e., *5-HTTLPR* S/S or S/L, *DRD4 VNTR* 7R, and additive risk) and two criterion variables (i.e., attachment security and CBCL-DP). Cumulative ACEs was entered at Block 3, followed by a G × E interaction term at Block 4.

Mplus 8.4 (Muthén & Muthén, 1998) was used to construct longitudinal panel models to test cascade effects. Full information maximum likelihood estimation was used to handle missing data (Enders, 2010). Goodness of fit for overall models was judged based on the following benchmarks: comparative fit index ≥ 0.95, Tucker-Lewis Index ≥ 0.95, root mean square error of approximation ≤ 0.06, and standardized root mean residual ≤ 0.08 (Hu & Bentler, 1999). Local fit testing using standardized path coefficients was used to estimate direct effects within models.

First, an autoregressive panel model including paths for all cross-sectional associations between constructs at each of the four time points (Y3, 5, 9, and 15) as well as all possible autoregressive terms was run. A random-intercept cross-lagged panel models (RI-CLPM) was then used to examine longitudinal and transactional associations among Attachment Security, Dysregulation, Negative Parenting, Social Competency, and LPF. RI-CLPMs have an advantage over CLPMs in that they account for time-invariant, trait-like stability as well as temporal stability and allow for the separation of within-person and between-person variance through the inclusion of random intercepts (Hamaker et al., 2015). Random intercepts are included in the model to anchor or mean center each individual’s across-time regression equation to predict observed scores on the key constructs, reflecting each individual’s stable traits (Hamaker et al., 2015). The RI-CLPM included all paths from the previous model as well as cross-lagged pathways to account for longitudinal associations between constructs at adjacent time points (e.g., Y3 Dysregulation → Y5 Negative Parenting). The total autoregressive effects and total cross-lagged effects across time were computed as unstandardized path coefficients.

A third and final model was run including all paths from previous models. In addition, Dysregulation, Negative Parenting, and Social Competency at age nine were modeled as proximal predictors of LPF at age 15. Indirect pathways from Attachment, Dysregulation, Negative Parenting, and Social Competency at age three to LPF at age 15 was also specified.

### Covariate data analyses

Given that some research suggests that sex (Dmitrieva et al., 2011; Gressier et al., 2016) and race (Gelernter et al., 1999) may moderate the expression of *5-HTTLPR* and *DRD4 VNTR*, prior to running regression analyses, preliminary analyses were run to determine whether this was the case in the present sample. Race and ethnicity (i.e., non-Hispanic white, non-Hispanic Black, Hispanic, other races), which was reported by mothers at baseline, was recoded into dichotomous dummy variables, with non-Hispanic whites serving as the reference category. Sex and the race dummy variables were entered at Block 1, and risk genotype, a genotype by sex interaction term, and genotype by race interaction terms were entered at Block 2. Results of all models showed that none of the interaction terms were significant, indicating that sex and race did not significantly moderate the impact of genotype on attachment security or dysregulation. As such, the interaction terms were not included in analyses for the sake of parsimony.

Prior to analyzing structural models, a multigroup analysis was run to examine potential sex differences. The autoregressive panel model was run first with all parameters constrained to be equal and next with all parameters free to vary across sexes. Results revealed significant differences between the constrained and unconstrained models (Δχ^2^ (18) = 59.63, *p* < .001). As such, models were run separately for males and females.

## Results

Table 2 contains descriptive statistics and bivariate correlations between predictor variables within each time point in males and females. Most of the sample (77%) reported at least one ACE, and 32.1% reported three or more ACEs, with 74.1% reporting at least one type of household dysfunction and 26.3% reporting at least one type of maltreatment. Parental mental health problems was the most common experience (54.5%), followed by parental separation (50.1%), domestic violence (35.8%), emotional or psychological abuse (31.8%), parental incarceration (23.8%), physical abuse (16.8%), parental substance abuse (15.2%), and neglect (10.7%).

### Effects of genotype and ACEs on attachment security

Results indicated that neither sex nor *5-HTTLPR* status (i.e., heterozygous with short allele or homozygous for short allele vs. homozygous for long allele) was significantly associated with attachment security (*β* = .041, *p* = .10 and *β* =.003, *p* = .89, respectively). However, ACEs was significantly associated with attachment security (*β* = −.095, *p* < .001) over and above sex and genetic risk (*βR* = .095, *F* change = 14.26, *p* < .001). Adding *5-HTTLPR* x ACEs at Block 4 did not incrementally predict attachment security (*F* change = .006, *p* = .94).

Similarly, neither sex nor *DRD4 VNTR* status were significant predictors of attachment security (*β* = .038, *p* = .12 and *β* = −.004, *p* = .86, respectively). At Block 3, ACEs was significantly associated with attachment security over and above sex and genetic risk (*β* = −.084, Δ*R* = .083, *F* change = 11.87, *p* < .001). Adding *DRD4 VNTR* x ACEs at Block 4 did not increment prediction of attachment security (*F* change = 1.44, *p* = .23).

Results examining additive genetic risk were similar. Neither sex nor additive genetic risk were significantly associated with attachment security (*β* = .038, *p* = .12 and *β* = .11, *p* = .15, respectively). At Block 3, ACEs was significantly associated with attachment security over and above sex and genetic risk (*β* = −.084, Δ*R* = .083, *F* change = 11.95, *p* < .001). Adding additive risk x ACEs at Block 4 did not increment prediction (*F* change = 1.49, *p* = .22).

### Effects of genotype and ACEs on dysregulation

Sex was a significant predictor of CBCL-DP scores, such that males tended to have higher dysregulation than females overall (*β* = −.065, *p* = .002). Race and ethnicity was also a significant predictor of CBCL-DP scores, with non-Hispanic Blacks, other races, and Hispanics reporting significantly higher levels of dysregulation relative to non-Hispanic Whites (*β*s = .18, .06, .11, respectively, *p* < .01).

At Block 2, *5-HTTLPR* status was a significant predictor of CBCL-DP scores over and above sex (*β* = −.050, Δ*R* = .045, *F* change = 5.47, *p* = .019); however, when ACEs was added at Block 3, genotype was no longer significant in the regression equation (*β* = −.026, *p* = .221). ACEs was significantly associated with CBCL-DP and resulted in a significant increment of variance in CBCL-DP predicted (*β* = .250, Δ*R* = .25, *F* change = 145.16, *p* < .001). Adding *5-HTTLPR* x ACEs at Block 4 did not significantly incrementally predict CBCL-DP scores (*F* change = 0.057, *p* = .81).

In contrast, *DRD4 VNTR* status was not a significant predictor of CBCL-DP scores (*β* = .016, *p* = .37). At Block 3, ACEs was significantly positively associated with CBCL-DP and resulted in a significant increment of variance (*β* = .25, Δ*R* = .25, *F* change = 223.28, *p* < .001). Adding *DRD4 VNTR* x ACEs at Block 4 did not increment prediction (*F* change = .96, *p* = .33).

Additive genetic risk was not a significant predictor of CBCL-DP scores (*β* = −.006, *p* = .73). ACEs was significantly positively associated with CBCL-DP and resulted in a significant increment of variance (*β* = .25, Δ*R* = .25, *F* change = 224.22, *p* < .001). Adding additive risk × ACEs at Block 4 did not incrementally predict dysregulation (*F* change = 0.32, *p* = .57).

### Post-Hoc analyses separating maltreatment and household dysfunction

Post-hoc analyses explored whether separating maltreatment or household dysfunction in the regression equation would reveal one of these components of ACEs as driving the above findings. Results indicated that maltreatment was more strongly associated with attachment security than household dysfunction; while maltreatment was a significant predictor of attachment security in both regression models (*β*s ranging from −.074 to −.082, *ps* < .01), household dysfunction was significant only in the regression model examining risk due to *5-HTTLPR* genotype (*β* = −.057, *p* = .02). Both maltreatment (*β* = .24) and household dysfunction (*β* = .13) were significant (*p* < .001) predictors of CBCL-DP.

### Cascade effects

**Figure 1.**
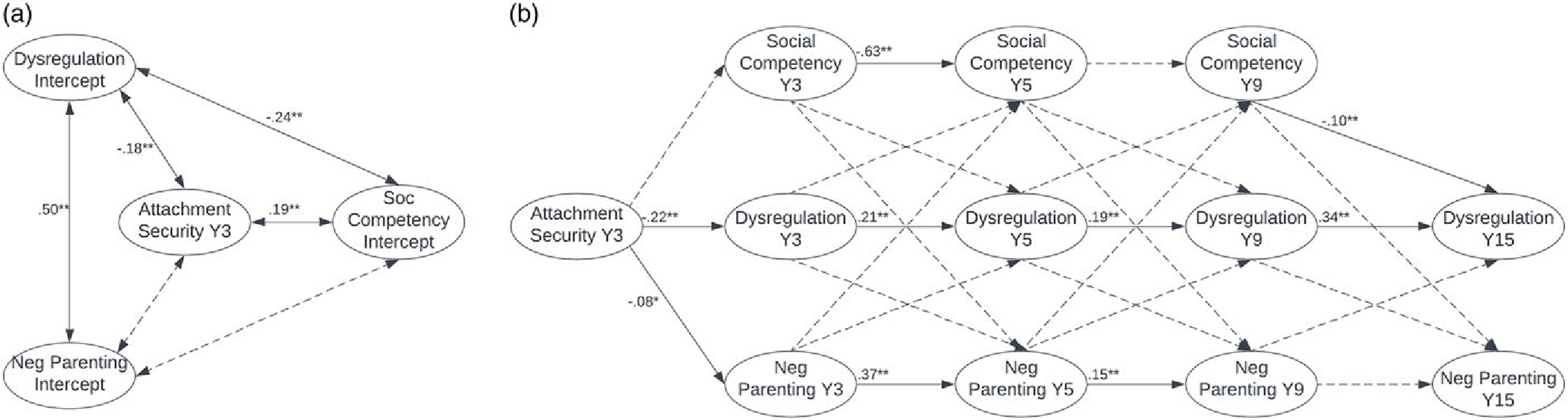
RI-CLPM for males with values displayed as standardized path coefficients. ** indicates significant at *p* < .01; * indicates significant at *p* < .05; dashed connecting line indicates not significant. Dysregulation = CBCL-DP; social = social competency; neg parent = negative parenting attitudes and behaviors; *Y* = year. Concurrent associations were computed but are not pictured. (***a***) Time-invariant effects. (***b***) Modeling changes across persons.

**Figure 2.**
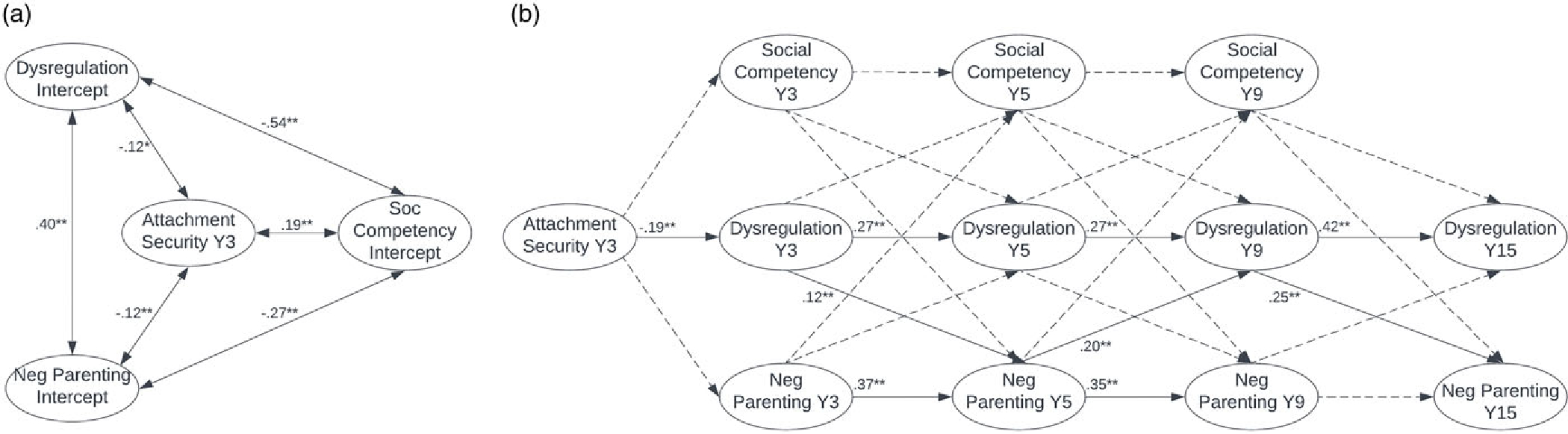
RI-CLPM for females with values displayed as standardized path coefficients. ** indicates significant at *p* < .01; * indicates significant at *p* < .05; dashed connecting line indicates not significant. Dysregulation = CBCL-DP; social = social competency; neg parent = negative parenting attitudes and behaviors; *Y* = year. Concurrent associations were computed but are not pictured. (***a***) Time-invariant effects. (***b***) Modeling changes across persons.

As shown in Table 3, the RI-CLPMs for males and females had good fit overall. For both males and females, fit statistics for the RI-CLPMs suggest that the addition of cross-lagged pathways improved fit relative to the autoregressive models. Figures 1 and 2 depict the RI-CLPMs for males and females, respectively, with values reported as standardized path coefficients. Cross-sectional associations between variables were computed but are not included in the figures for the purpose of readability.

As shown in Figure 1a, among males, Y3 attachment security was a significant predictor of time-invariant, between-persons levels of dysregulation and social competency. Attachment security was not significantly associated with the negative parenting intercept. Dysregulation was significantly associated with the negative parenting and social competency intercepts. After partialling out time-invariant effects and occasion-specific mean scores and adding cross-lagged paths to the model, Y3 attachment security remained a significant concurrent predictor of degree of deviation in Y3 dysregulation and negative parenting among males (see Fig. 1b). Local path coefficients within autoregressive paths for dysregulation were positive and significant, as were local path coefficients within autoregressive paths for negative parenting, except between ages nine and fifteen. Inconsistent with hypotheses, social competency did not exhibit this pattern. Rather, degree of deviation in Y3 social competency was a significant negative predictor of degree of deviation in Y5 social competency, and degree of deviation in Y5 social competency was not significantly associated with degree of deviation in Y9 social competency.

Total autoregressive effects for dysregulation, negative parenting, and social competency were significant for males (*b*_*CBCL-DP*_ = 0.44, *b*_*NegPar*_ = 0.79, *b*_*SocComp*_ = 1.19, all *p* < .01). Although local path coefficients for individual cross-lagged pathways were not significant, the total cross-lagged effects of dysregulation on negative parenting (*b*_*CBCL-DP on NegPar*_ = 0.40, *p* = .046), dysregulation on social competency (*b*_*CBCL-DP on SocComp*_ = −0.10, *p* = .01), and social competency on dysregulation (*b*_*SocComp on CBCL-DP*_ = −0.05, *p* = .006) were significant. Stated differently, there were significant within-person differences among males such that higher dysregulation in males predicted lower social competency and more negative parenting later in development, and lower social competency in males predicted greater dysregulation later in development.

As shown in Figure 2a, among females, Y3 attachment security was a significant predictor of time-invariant, between-persons levels of dysregulation, negative parenting, and social competency. Associations among dysregulation, negative parenting, and social competency were also significant.

After partialling out time-invariant effects and occasion-specific mean scores and adding cross-lagged paths to the model, Y3 attachment security remained a significant concurrent predictor of degree of deviation in dysregulation (see Fig. 2b). Local path coefficients within autoregressive paths for dysregulation were positive and significant, as were local path coefficients within autoregressive paths for negative parenting, except between ages nine and fifteen. Local path coefficients within cross-lagged paths indicated that greater deviation in Y3 and Y9 dysregulation predicted greater deviation in negative parenting attitudes and behaviors at Y5 and Y15, respectively. More deviation in Y5 negative parenting also predicted greater deviation in Y9 dysregulation.

Total autoregressive effects for dysregulation and negative parenting, and not social competency, were significant for females (*b*_*CBCL-DP*_ = 0.32, *b*_*NegPar*_ = 0.59, *ps* < .01). The total cross-lagged effects of dysregulation on social competency (*b*_*CBCL-DP on SocComp*_ = −0.17, *p* < .001) and negative parenting on social competency (*b*_*NegPar on SocComp*_ = −0.02, *p* = .025) were significant. This indicates that there were significant within-person differences among females, such that higher dysregulation and more negative parenting predicted poorer social competency later in development. The total cross-lagged effect of dysregulation on negative parenting neared significance (*b*_*CBCL-DP on NegPar*_ = 0.33, *p* = .09).

### Prediction of LPF

Overall model fit remained good when LPF was added to the model as an endogenous variable predicted by Y9 dysregulation, negative parenting, and social competency (see Table 3).

**Figure 3.**
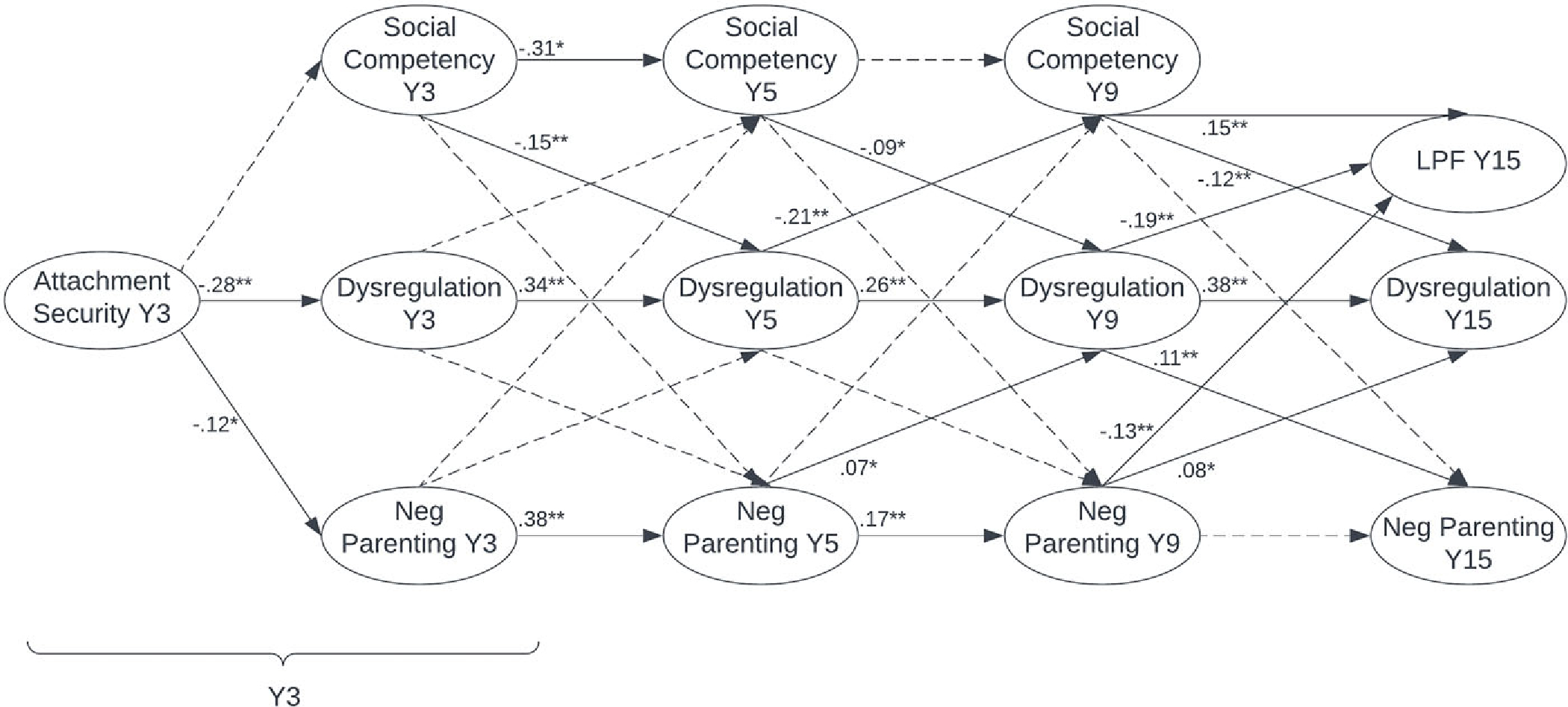
RI-CLPM with cascades to LPF for males. ** indicates significant at *p* < .01; * indicates significant at *p* < .05; dashed connecting line indicates not significant. Dysregulation = CBCL-DP; social = social competency; neg parent = negative parenting attitudes and behaviors; *Y* = year; LPF = level of personality functioning. Concurrent associations were computed but are not pictured.

As shown in Figure 3 and consistent with the previous model, Y3 attachment security remained a significant concurrent predictor of degree of deviation in dysregulation and negative parenting among males. Local path coefficients within autoregressive paths for dysregulation, negative parenting, and social competency were also consistent with results of the previous model.

Local path coefficients for some cross-lagged paths also emerged as significant. Specifically, lower deviation in Y3, Y5, and Y9 social competency predicted higher deviation in Y5, Y9, and Y15 dysregulation, respectively. Higher deviation in Y5 and Y9 negative parenting also predicted higher deviation in Y9 and Y15 dysregulation, respectively. Greater deviation in Y5 dysregulation predicted lower deviation in Y9 social competency, and greater deviation in Y9 dysregulation predicted more deviation in Y15 negative parenting.

There were significant direct effects of deviation in Y9 dysregulation, negative parenting, and social competency on Y15 LPF among males (see Fig. 3). Specifically, local path coefficients showed that 15-year-old males with greater deviation in dysregulation and negative parenting and lower deviation in social competency tended to have less adaptive personality functioning (i.e., lower LPF). There were also significant indirect effects of Y3 dysregulation and negative parenting on LPF, with greater Y3 dysregulation (*β* = −.033, *p* < .001) and more Y3 negative parenting (*β* = −.015, *p* = .031) predicting lower Y15 LPF. A significant indirect effect of attachment on LPF was also observed, such that greater Y3 attachment security predicted more adaptive personality functioning (i.e., higher LPF) at age 15 via lower emotion dysregulation and higher social competency across childhood (*β* = .011, *p* < .001).

**Figure 4.**
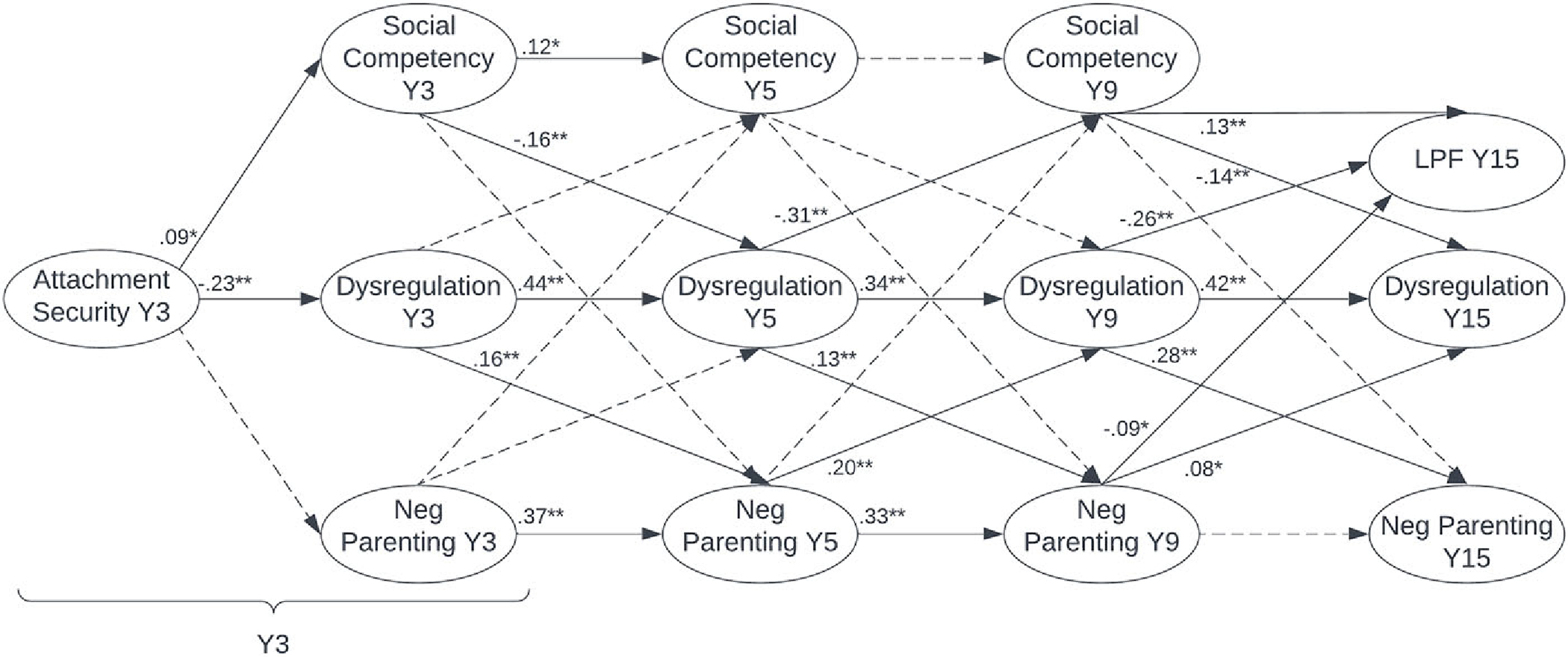
RI-CLPM with cascades to LPF for females. ** indicates significant at *p* < .01; * indicates significant at *p* < .05; dashed connecting line indicates not significant. Dysregulation = CBCL-DP; social = social competency; neg parent = negative parenting attitudes and behaviors; *Y* = year; LPF = level of personality functioning. Concurrent associations were computed but are not pictured.

As shown in Figure 4, among females, Y3 attachment security remained a significant concurrent predictor of deviation in dysregulation and also emerged as a significant concurrent predictor of deviation social competency. As with the previous model, local path coefficients within autoregressive paths for dysregulation, negative parenting (expect between ages nine and 15), and social competency (except between ages five and nine) were positive and significant. Local path coefficients within cross-lagged paths indicated that greater deviation in Y3, Y5, and Y9 dysregulation predicted more deviation in Y5, Y9, and Y15 negative parenting, respectively. Greater deviation in Y5 dysregulation predicted less deviation in Y9 social competency. Finally, less deviation in Y3 and Y9 social competency predicted higher deviation in Y5 and Y15 dysregulation, respectively.

Among females, deviation in Y9 dysregulation, negative parenting, and social competency had significant direct effects on LPF at age 15 (see Fig. 4). Specifically, local path coefficients showed that 15-year-old females with greater deviation in dysregulation and negative parenting and lower deviation in social competency tended to have less adaptive personality functioning (i.e., lower LPF) at age 15. There were significant indirect effects of Y3 dysregulation, negative parenting, and social competency on LPF, with greater Y3 dysregulation (*β* = −.076, *p* < .001), more Y3 negative parenting (*β* = −.037, *p* < .001), and poorer Y3 social competency (*β* = .031, *p* < .001) predicting lower Y15 LPF. A significant indirect effect of attachment on LPF was also observed, such that greater Y3 attachment security predicted more adaptive personality functioning (i.e., higher LPF) at age 15 via lower emotion dysregulation and less negative parenting across childhood (*β* = .021, *p* < .001). Indirect effects via pathways encompassing social competency were not significant.

## Discussion

The present research examined etiological processes involved in personality development across early and middle childhood within a developmental cascades framework. Following Masten and Cicchetti’s (2010) call for the need for longitudinal data collection across multiple levels of analysis, this project incorporated genetic variables as well as psychological and interpersonal constructs measured at multiple time points. Importantly, data included prospective assessments of ACEs within the first three years of life, enabling examination of the predictive impact of environmental risk during this critical period. Moreover, the sample utilized was comprised of low-income urban families, which allowed for examination of the impact of proximal processes on individual differences in conditions of greater instability and adversity.

### Genetic and environmental impact on attachment and regulation

Contrary to hypotheses, neither risk genotype (i.e., *5-HTTLPR* and *DRD4 VNTR* status) nor the genetic risk × ACEs interaction terms were significant predictors of attachment security at age three. Rather, results revealed that ACEs was a significant predictor of attachment security at age three over and above sex or genetic risk, as assessed by *5-HTTLPR* and *DRD4 VNTR* status. Of note, in a post-hoc analysis separating maltreatment and household dysfunction, results suggested that maltreatment may be a more important predictor of attachment security than household dysfunction when examined in relation to genetic risk.

Consistent with some previous research (e.g., Mitchison et al., 2020), sex was a significant predictor of dysregulation, such that males tended to have higher dysregulation scores than females. Aside from this, similar to findings for attachment, *DRD4 VNTR* status was not a significant predictor of dysregulation at age three. Although *5-HTTLPR* status was a significant predictor of dysregulation over and above sex, when ACEs was added to the regression equation, *5-HTTLPR* status was no longer significant. Rather, ACEs was a significant predictor of dysregulation at age three, over and above sex and genetic risk. The genetic risk × ACEs interaction terms did not significantly incrementally predict dysregulation for any of the risk genotypes.

Although inconsistent with hypotheses, these findings do appear consistent with assumptions of Bronfenbrenner’s bioecological theory, which predicts that heritability varies as a joint function of proximal processes and the environment and that stronger proximal processes enable higher heritability (Bronfenbrenner & Ceci, 1994). It follows that the proportion of individual differences attributable to actualized genetic potential is maximized when environmental risk is minimized. Conversely, as level of environmental risk increases, genetic factors become relatively less important, and adverse experiences become stronger determinants of given outcomes (Bronfenbrenner & Morris, 2006). Findings of the present study are consistent with this model in that the genetic polymorphisms examined were relatively less important, and adverse childhood experiences were relatively more important, in predicting adverse outcomes in this high-risk sample.

### Relationships among attachment, dysregulation, negative parenting, and social competency

Results of cascade models were partially consistent with hypotheses. Overall, models demonstrated good fit, and, importantly, the addition of cross-lagged paths improved model fit, indicating that data were better explained by models accounting for transactions among variables across time than by a model accounting only for temporal consistency of variables.

Among males and females, higher attachment security at age three was associated with stable differences in key constructs over time. Specifically, males with higher attachment security at age three tended to also display less dysregulation and higher social competency across childhood. Females with higher attachment security at age three tended to also display less dysregulation and higher social competency and to have caregivers with less negative parenting attitudes and behaviors across childhood. Both males and females with consistently poorer regulation over time also tended to be consistently less socially competent and to have caregivers with stable, more negative parenting attitudes and behaviors. These findings are consistent with attachment theory and further support the idea that self-regulatory capacity develops within the context of early caregiving environment (e.g., Sroufe, 2005).

Consistent with hypotheses, total autoregressive effects across time indicated that dysregulation and negative parenting exhibited temporal stability among both males and females. Findings also showed significant cross-sectional correlations between these variables in males and females at multiple timepoints. Stated differently, children with greater deviation in emotional and behavioral dysregulation also had caregivers who reported more deviation in negative parenting attitudes and behaviors at the same timepoint.

Findings also partially supported hypotheses concerning evocative interaction effects among dysregulation, negative parenting, and social competency. Among males, findings showed that higher deviation in dysregulation predicted greater deviation in negative parenting later in development. Unexpectedly, higher deviation in dysregulation also predicted lower deviation in social competency later in development among males, and lower deviation in social competency predicted greater deviation in dysregulation later in development. Similarly, among females, higher deviation in dysregulation and negative parenting predicted less deviation in social competency later in development. Although other cross-lagged effects did not reach significance, these findings still provide broad support for cascading relationships among self-regulation, caregiving environment, and interpersonal skills and suggest that development in each of these areas may have important implications for development in other areas.

### Cascades to LPF

Overall model fit of RI-CLPMs for males and females remained good when LPF was added as an endogenous variable. Consistent with hypotheses, among males and females, greater deviation in emotional and behavioral dysregulation and negative parenting and lower deviation in social competency at age nine predicted less adaptive personality functioning at age 15. Among males, greater attachment security at age three predicted more adaptive personality functioning via lower emotion dysregulation and higher social competency. Among females, however, greater attachment security at age three predicted more adaptive personality functioning via lower emotion dysregulation and less negative parenting attitudes and behaviors across childhood. That deviation in dysregulation was significantly negatively associated with LPF is consistent with research on the development of personality pathology showing that emotional and behavioral dysregulation are predictive of development of personality pathology (e.g., Stepp et al., 2016). The negative association between deviation in negative parenting and LPF among females is also consistent with previous research findings showing that close relationships, such as relationships with caregivers, play important roles in the development of self and interpersonal functioning (Ainsworth, 1989; Kaufman & Crowell, 2018).

It should also be noted that the strength of the association between LPF and dysregulation relative to associations with negative parenting or social competency, is likely due in part to shared method variance. Although individual items themselves did not overlap, both dysregulation and the parent-report indicators of LPF were derived from the CBCL. Because the systematic error of method variance was aligned in this case, the association is stronger than it would be for two independently measured variables (Meyer et al., 2001).

### Limitations and future research directions

Access to the FFCWS datasets substantially increased the feasibility of this multimethod, longitudinal study; however, the use of archival data also limited control over measure selection and study methodology in at least three key ways. First, the full CBCL was not administered at each time point due to time constraints, and, as a result, not all items contributing to CBCL-DP were available for all time points. The items included were weighted more heavily towards aggressive behaviors, and relatively smaller proportions of the Anxious/Depressed and Attention subscales were included. Similarly, items assessing social competency were adapted from the ASBI. Although the number of items remained consistent across timepoints, the wording of items intended to reflect the same behavior differed across different timepoints, with the greatest differences in wording at age nine. Additionally, these items were completed by mothers at each time point, as opposed to by peers or other observers (e.g., teachers) who may have had more opportunities to witness the focal child’s social interactions. This may have, in part, accounted for the unexpected patterns of Social Competency in longitudinal models.

Second, the number of items relevant to each ACE varied. To address this, between-subjects *z*-scores were computed for each ACE category, and cumulative ACEs was calculated as the mean of these *z*-scores. This method had the advantage of making use of all available data; however, it also makes it difficult to derive descriptive data and to compare ACEs in this sample to other samples. Cumulative ACEs were also computed using parent-report at ages one and three. Due to the nature of the constructs being assessed, it seems feasible that some parents would be hesitant to report honestly and may have under-reported problems as a result.

Third, the measures selected were those that most closely aligned with the constructs of interest; however, certain constructs may have been better captured through other measures or data collection methods. Most notably, the study did not include a validated measure of LPF or personality functioning. Rather, a measure was derived from items with content that was deemed to best reflect aspects of LPF. The resultant variable was weighted more heavily towards interpersonal functioning than towards identity and self-functioning, which may not optimally reflect LPF as a construct (Bender et al., 2011). Moreover, data collected at birth through age 9 were parent- or caregiver-report; however, data collected at age 15 was primarily self-report by the focal child. As such, caregiver-report measures made up the majority of the data included in the model; not only did this result in little independence in data sources over time, but, because endogenous predictors of LPF were caregiver-report, including self-report at age 15 introduced heteromethod variance into the model, which may have limited the extent to which data could optimally fit the model (Meyer et al., 2001). The inclusion of youth self-report where possible is consistent with best practice in assessing youth psychopathology, which suggests including reports from multiple informants (in this case, parent and child) across multiple contexts and perspectives in order to enhance prediction (De Los Reyes et al., 2023). Nonetheless, research also indicates that the selection of informants and the manner in which multiple reports are integrated is important (Makol et al., 2020), and it may be that including multiple informants (e.g., parent, teacher, peer, self) reports’ over time would yield better prediction.

Finally, this study operationalized genetic risk based on polymorphisms of the serotonin transporter and dopamine D4 receptor genes. These candidate genes were selected due to their documented relationships with attachment and emotion dysregulation and availability in the dataset; however, they do not reflect all possible candidate genes that have been examined in relation to these constructs. Moreover, there are notable limitations of examining candidate genes rather than taking a genome-wide approach, and the potential of genome-wide studies for increasing understanding of genetic contributions to psychological issues is arguably far greater than that of candidate gene studies (Duncan et al., 2019).

### Future research directions

Researchers have noted that ACEs encompass a definition of adversity that focuses on an individuals’ immediate home environment and does not directly account for social determinants of health and other social factors (McEwen & Gregerson, 2019). The present research took steps towards addressing social factors by utilizing data from a high-risk sample of low socioeconomic status; however, future research may wish to expand on this by utilizing a sample with greater variability in level of environmental risk or by directly accounting for potential effects of social factors within statistical models. Doing so would allow for examination of whether and the extent to which the relative impacts of genetics and environment on attachment and dysregulation are dependent on context. The present research also included cumulative ACEs only over the first three years of life to test predictive effects of early childhood exposure to adversity. It may also be useful to incorporate maltreatment and household dysfunction ACEs into cascade models at each data collection wave to examine the impact of types of environmental risk on the constructs of interest over time. For instance, research on child maltreatment has demonstrated that the timing at which maltreatment occurs, in addition to its severity and chronicity, is important for understanding the effects of maltreatment, and has demonstrated that resilience is associated with different factors in maltreated versus non-maltreated children (e.g., Cicchetti, 2013). Based on prior research, it may be that social competency is a more important predictor of adaptive outcomes for non-maltreated children, whereas self-regulation is a more important predictor for maltreated children (Cicchetti, 2013).

Future research may also explore genetic contribution to attachment security and dysregulation in different ways. Researchers (Leerkes et al., 2017; Picardi et al., 2020) have noted that findings of molecular genetic studies have been inconsistent, and some assert that genome-wide analyses yield more useful information (Duncan et al., 2019). As such, future research may wish to explore emotion regulation with respect to the full genome. Additionally, a growing body of research suggests that ACEs may be associated with epigenetic changes and alterations in telomere length (Lang et al., 2019). It would be beneficial for future research to examine potential associations of ACEs with epigenetic alterations in serotonin or dopamine functioning. In addition, racial or ethnic differences could be examined, as previous research has shown variation in serotonin functioning across racial groups (Gelernter et al., 1999).

The limitations posed by use of archival data could be addressed in future research by exploring similar research questions using alternative measures and data collection methods. As discussed above, because children’s functioning often varies by context and because agreement of multiple informants is typically low to moderate (e.g., Achenbach et al., 1987), best practices for child assessment involves collecting data from multiple informants, such as parents, teachers, peers, and children themselves, or through multiple methods, such as self- and informant-reports, behavioral tasks, and real-world outcomes (Achenbach & Rescorla, 2015). Researchers also recommend that, to the extent possible, item content and scaling is held constant across measures being completed by multiple informants (De Los Reyes et al., 2015) and that measures are held constant across data collection timepoints (Achenbach & Rescorla, 2015). Tools such as the ASEBA instruments (Achenbach & Rescorla, 2001), which have parallel parent- and teacher-report forms for ages six through 18 and a parallel self-report form for ages 11 though 18, facilitate such research. Future research could utilize all CBCL items and could incorporate the ASEBA Teacher Report Form as well as the Youth Self Report when children reach an appropriate age.

In addition, the present study operationalized negative parenting attitudes and behaviors as a composite derived from four items related to parenting attitudes and 15 items related to maltreatment. Although maltreatment has been shown to predict outcomes such as poorer self-regulation and higher levels of psychopathology (Kim & Cicchetti, 2010), the base rate of these behaviors is likely much lower than that of less severe, but still potentially detrimental parenting behaviors, such as lack of responsivity or invalidation of children’s emotions. It also seems feasible that positive parenting practices and other positive life experiences could act as protective factors that would facilitate optimal development. As such, future research incorporating other relevant parenting practices, including positive parenting practices that could protect against adverse outcomes, would be beneficial.

RI-CLPMs effectively model cascading relationships between variables, and cross-lagged relationships in these models capture within-person changes after accounting for group means and individual deviation from these means. Usami (2019) notes that applying RI-CLPMs is a reasonable choice for inferring reciprocal relationships between variables, as most causal relations in behavioral sciences are typically viewed as operating at the within-person level. However, RI-CLPMs do not explicitly model developmental trajectories (Usami et al., 2019). Thus, future research could implement latent curve models or variations in these models to better capture group- and individual-level differences in developmental trajectories.

Finally, it would be beneficial for future research to utilize alternative methods for assessing LPF. For example, previous research has assessed LPF using self-report measures (Waugh et al., 2020) and semi-structured interviews (Weekers et al., 2021). Alternatively, future research could operationalize LPF as a composite derived from multiple data sources, such as observational or life history data, performance-based measures and self- and informant-report.

## Conclusion

Findings of the present research contribute to the developmental psychopathology literature by highlighting the significant impact of early exposure to environmental risk factors on social and emotional development. Importantly, this study expanded upon the well-documented impact of ACEs on a variety of adverse outcomes (e.g., Felitti & Anda, 2010) by examining the relative impacts of genetics, ACEs, and G × E interactions on early development and suggesting that, consistent with Bronfenbrenner’s hypothesis (Bronfenbrenner & Morris, 2006), in this high-risk sample, genetic factors were relatively less important, and environmental risk had a stronger impact on key outcomes.

Results of cascade models showed that, among males, greater early childhood attachment security predicted more adaptive personality functioning via lower emotion dysregulation and higher social competency. Among females, greater attachment security at age three predicted more adaptive personality functioning via lower emotion dysregulation and less negative parenting attitudes and behaviors across childhood. These findings help elucidate longitudinal relationships from early childhood attachment security to adolescent personality functioning as well as contributions of emotion dysregulation, parenting, and social competency to personality development.

## Figures and Tables

**Table 1.** Measures and corresponding respondents across data collection waves

Wave	Y0	Y1	Y3	Y5	Y9	Y15
Retention rate		89%	86%	85%	74%	73%
Age of focal child	Birth	1 year	3 years	5 years	9 years	15 years
Demographics	M	M				
Adverse Childhood Experiences		M	M			
Toddler Attachment Q-Sort			I			
Child Behavior Checklist			M	M	M	M^a^
Adaptive Soc Beh Inventory ES			M	M	M	C^a^
Conflict Tactics Scale–Parent-Child			M	M	M	M
Aggravation in Parenting			M	M	M	M
EPOCH Wellbeing						C^a^
Social Skills Rating System AS						C^a^
Level of Personality Functioning (LPF)						M, C
DNA collection					I	

*Note.* M = mother or primary caregiver; I = interviewer at in-home assessment; C = focal child.

a items from these measures were used to derive LPF. Adaptive Soc Beh Inventory ES = Adaptive Social Behavior Inventory Express Scale; AS = Assertion Scale.

**Table 2.** Descriptive statistics and bivariate correlations among primary variables across time points in males and females

	*M (SD)*	1	2	3	4	5	6	7	8	9	10	11	12
Males (*N* = 1165)													
1. Attach	−1.6 (1.1)												
2. CBCL-DP Y3	5.8 (5.6)	**−.27**											
3. Soc Competency Y3	15.1 (2.8)	**.15**	−.05										
4. Neg Parenting Y3	0.06 (0.8)	**−.11**	**.28**	*−.07*									
5. CBCL-DP Y5	3.9 (4.1)	**−.16**	**.45**	**−.09**	**.20**								
6. Soc Competency Y5	15.5 (2.8)	**.10**	−.06	**.31**	−.06	.00							
7. Neg Parenting Y5	0.05 (0.8)	**−.08**	**.18**	*−.07*	**.58**	**.26**	−.06						
8. CBCL-DP Y9	11.9 (10.4)	**−.14**	**.29**	*−.07*	**.16**	**.34**	**−.09**	**.17**					
9. Soc Competency Y9	24.0 (4.9)	**.14**	**−.16**	**.32**	−.06	**−.16**	**.33**	−.04	**−.20**				
10. Neg Parenting Y9	0.04 (0.8)	−.05	**.17**	−.04	**.41**	**.15**	*−.07*	**.44**	**.32**	**−.09**			
11. CBCL-DP Y15	6.20 (5.9)	**−.08**	**.28**	−.05	**.18**	**.31**	−.04	**.20**	**.46**	**−.18**	**.27**		
12. Neg Parenting Y15	0.06 (0.8)	−.05	**.16**	−.01	**.34**	**.17**	.03	**.37**	**.17**	−.04	**.43**	**.40**	
13. LPF	0.02 (0.8)	**.14**	**−.20**	**.11**	**−.14**	**−.16**	**.11**	**−.12**	**−.26**	**.22**	**−.20**	**−.61**	**−.29**
Females (*N* = 1103)													
1. Attach	−1.5 (1.0)												
2. CBC-DP Y3	5.1 (5.2)	**−.22**											
3. Soc Competency Y3	15.5 (2.6)	**.12**	*.07*										
4. Neg Parenting Y3	−0.06 (0.8)	*−.07*	**.30**	−.01									
5. CBCL−DP Y5	3.5 (3.7)	**−.13**	**.48**	*−.08*	**.19**								
6. Soc Competency Y5	15.9 (2.4)	.03	−.03	**.18**	−.01	**.14**							
7. Neg Parenting Y5	−0.06 (0.8)	*−.07*	**.27**	−.05	**.60**	**.32**	.00						
8. CBCL-DP Y9	9.4 (8.9)	**−.12**	**.31**	−.02	**.20**	**.42**	.01	**.29**					
9. Soc Competency Y9	24.8 (4.5)	**.12**	**−.18**	**.15**	**−.11**	**−.21**	**.15**	**−.12**	**−.24**				
10. Neg Parenting Y9	−0.04 (0.8)	**−.12**	**.24**	*−.07*	**.47**	**.23**	−.04	**.58**	**.42**	**−.16**			
11. CBCL-DP Y15	5.8 (6.1)	−.03	**.22**	−.06	**.20**	**.37**	−.03	**.26**	**.51**	**−.21**	**.30**		
12. Neg Parenting Y15	−0.06 (0.8)	−.06	**.16**	−.04	**.37**	**.20**	−.04	**.44**	**.30**	**−.11**	**.47**	**.44**	
13. LPF	−0.03 (0.8)	.05	**−.17**	**.11**	**−.13**	**−.29**	.05	**−.19**	**−.34**	**.20**	**−.21**	**−.65**	**−.32**

*Note.* Bold font indicates correlation is significant at *p* < .01; italicized font indicates correlation is significant at *p* < .05.

**Table 3.** Fit statistics for cascade models in males and females

Model	*CFI*	*TLI*	*RMSEA [90% CI]*	SRMR
Males				
AR Panel	.948	.916	.055 [.047, .062]	.058
RI-CLPM	.985	.958	.036 [.024, .048]	.031
RI-CLPM with LPF	.968	.926	.049 [.040, .058]	.049
Females				
AR Panel	.947	.919	.054 [.046, .063]	.059
RI-CLPM	.987	.962	.037 [.025, .049]	.029
RI-CLPM with LPF	.981	.953	.041 [.031, .051]	.037

*Note.* AR = autoregressive; RI-CLPM = Random-Intercepts Cross-Lagged Panel Model; LPF = Level of Personality Functioning.
